# First Record of the Edible Mushroom *Lepista sordida* in Western Algerian Forest: Nutritional Value and Physicochemical Parameters of Mycelial Culture

**DOI:** 10.3390/jof9080858

**Published:** 2023-08-17

**Authors:** Yousra Alim, Warda Sidhoum, Soulef Dib

**Affiliations:** 1 Laboratoire de Biologie des Microorganismes et Biotechnologie, Faculté des Sciences de la Nature et de la Vie, University Oran 1, Es Senia 31100, Algeria; sidhoumwarda@yahoo.fr; 2 Département de Biologie, Université de Mostaganem Abdel Hamid Ibn Badis, Mostaganem 27000, Algeria

**Keywords:** sporophores, mycelium, saprophytic basidiomycete, optimal growth conditions, mushroom characterization, nutritional analysis

## Abstract

The exploration of the western forests of Algeria led to the remarkable discovery of the first occurrence of *Lepista sordida*, an edible wild mushroom of significant culinary importance for the local community, traditionally consumed in its natural state. This discovery was made possible through the use of various methods, including macroscopic observations (revealing a violet color) as well as microscopic observations conducted using scanning electron microscopy (SEM), revealing a cylindrical shape with distinct contours. Additionally, molecular analyses were conducted. Genomic DNA was extracted from the mycelium, followed by DNA amplification using specific primers targeting the internal transcribed spacer region (ITS1 and ITS2). After PCR reactions and sequencing of the obtained amplicons, the nucleotide sequences of the mycelium were submitted to the GenBank database of NCBI with the assigned accession number: MZ928450.1. These sequences were subsequently used to construct the phylogenetic tree. Furthermore, an in-depth study of physicochemical parameters was undertaken to determine the optimal conditions for cultivating the mycelium of this edible wild mushroom, including pH, temperature, relative humidity, and light. Different temperatures were examined: 20, 25, 30, 35, 40, and 45 °C. The effect of pH on mycelium growth was studied using a PDA agar medium with buffered values of 4, 5, 5.6, 6, 7, and 8. Similarly, six levels of relative humidity were tested: 14, 50, 74, 80, 95, and 100%. A study on the impact of light on mycelium growth was conducted by exposing Petri dishes inoculated with PDA to a light intensity of 500 lux for 5, 10, 15, 20, and 24 h. The results clearly demonstrated that variations in these different physicochemical parameters significantly influenced mycelium growth. For the *Lepista sordida* strain, growth was favored at pH levels of 4, 5, 6, and 6, with no growth observed at pH 7 and 8. The optimal temperature range for mycelium growth of *Lepista sordida* was 20–25 °C, while no growth was observed at 30, 35, 40, and 45 °C. Relative humidity levels of 74, 80, and 95% showed no significant differences. Optimization of mycelium growth and primordia production in *Lepista sordida* were successfully achieved. Optimal conditions for the primordia phase were identified as 25 °C, with humidity ranging from 90 to 95%. A nutritional analysis of fresh sporophores was conducted using established analytical methods. Notably, the nutritional composition of *Lepista sordida* sporophores exhibited high significance for the following parameters: moisture content (67.23 ± 1.90%), ash content (9.35 ± 0.66%), fat content (3.25 ± 0.24%), protein content (17.22 ± 0.38%), and carbohydrate content (63.83 ± 1.23%).

## 1. Introduction

Mushrooms have a long history that dates back long before the emergence of the human species, over a billion years ago, marking their divergence from the animal kingdom [1]. Biodiversity encompasses the variety of and variation in all living beings. Among these organisms, fungi hold a special place as one of the largest and most diverse groups on the planet [2]. Extensive research has been carried out in Africa, Algeria included, to investigate the diversity of fungi, particularly macromycetes (higher fungi), with the aim of gaining a deeper understanding of the extensive array of species and variations found within the fungal kingdom [3,4,5]. These studies have underscored the vital significance of fungi as a fundamental element of biodiversity.

The identification of filamentous fungi is based on cultural criteria and morphological criteria that combine the macroscopic appearance of cultures with microscopic morphology. In addition to cultural criteria such as temperature and growth rate, physiological criteria such as the study of sugar assimilation as carbon and energy sources are also considered [6].

Molecular data have greatly contributed to the identification of fungi. Molecular biology techniques, such as sequencing the ribosomal RNA gene, have been developed to determine the phylogenetic relationships among genera. Due to genetic variability, internal transcribed spacer (ITS) sequences provide better resolution at the species or subspecies level [7]. Wild mushrooms are a vital source of income and nutrition for many poor communities and are highly valued by recreational gatherers [8]. The consumption of wild mushrooms is widespread in Africa, but there is significant research activity regarding the beneficial properties of mushrooms [9].

Mushrooms have been commonly consumed since ancient times. The ancient Greeks believed that mushrooms were a source of strength for warriors in battle; they considered them the “food of the gods” and served them only on festive occasions [10]. Cultures also regard mushrooms as a nutritious food, often referred to as the “elixir of life” [11]. Edible wild mushrooms are non-timber forest products that play a crucial role in the dietary habits of rural populations and contribute to their overall wellbeing [12]. Mushrooms are used in food fortification formulations as sources of minerals and proteins. Their consumption holds great importance in nutrition due to their nutritional richness [13].

*Lepista* is a genus within the Tricholomataeae family that was established by Smith in 1870 and consists of approximately 50 species [14]. *Lepista* is widely distributed and encompasses several edible species, including *L. sordida*, *L. nuda*, and *L. saeva*. These mushroom species are relatively well known in Europe, America, and Australia, but are uncommon in Africa, except for Nigeria and South Africa [15].

The edible mushroom *Lepista sordida* is a producer of chemical compounds known as “fairy chemicals” or FCs (specific compounds produced by certain fungi, often associated with fairy rings and other environmental interactions). These substances are produced by its mycelium and find applications in agriculture, cosmetics, and medicine [16]. Additionally, the presence of metal ions, notably iron, in *Lepista sordida* holds significant physiological importance, contributing to essential activities such as transmitting bioelectronic signals, regulating biochemical reactions, and forming active enzyme centers within humans [17].

The goal of this study is to determine the species of edible mushroom collected in the western Algerian forest using molecular biology methods to evaluate their nutritional composition and to examine their specific mycelial growth conditions.

## 2. Materials and Methods

### 2.1. Collection of the Edible Mushroom

The wild mushroom was collected in the Tlemcen forest (35°07′41″ N 1°44′08.5″ W), situated in western Algeria. Various equipment was used for sampling, including a digital camera and plastic containers to preserve the fresh sporophores of the wild mushroom. These samples were promptly transported to the laboratory to prevent any degradation. The identification of the species was based on the examination of macroscopic and microscopic characteristics of the sporophores, as well as genetic characterization of the mycelium. Certain characteristics were recorded immediately, such as color and the presence or absence of a ring or a volva.

### 2.2. Morphological Characterization of the Mushroom

The species descriptions were based on the collection of sporophores that were conserved in an herbarium. The macroscopic and microscopic characteristics of fresh mushroom were studied following the methods described by [18,19].

Macroscopic characteristics were examined using both the naked eye and a Leica stereomicroscope, enabling the observation of various parts of the sporophore, such as the hymenophore, pileus, and lamellae. Measurements were recorded in centimeters (cm). Detailed descriptions were provided for the shape and color of various mushroom components, including the cap and hymenophore color, type of hymenophore, and arrangement of the lamellae.

Microscopic analysis of the spores, including their color, shape, and dimensions, was performed using an Olympus CX22 optical microscope manufactured in Tokyo, Japan. Scanning electron microscopy was used to examine the spores in greater detail, utilizing an analytical scanning electron microscope (JSM-6610LA manufactured in Japan) where an electron beam scanned the surface of the spores. According to the methodology described by [20], the samples were hydrated overnight at 48 °C then coated with gold for 45 s before being observed under a scanning electron microscope (SEM). The length of the hyphae as well as over 100 spores were measured using the graduated scale present in the microscope eyepiece, known as the “micrometer eyepiece”. Each diameter was measured at least 3 times, and the average of the measurements was used for calculations.

### 2.3. Isolation of the Mycelium

Potato dextrose agar (PDA) is the most recommended culture medium for studying physico-chemical characteristics as it promotes fungal growth and adaptation [21]. The mycelium of *Lepista* was isolated from the spore suspension. At first, the sporophore was disinfected with 70% ethanol for 3 min, followed by rinsing with sterile distilled water. It was then aseptically placed in a tube containing sterile distilled water and homogenized using an Ultra Turrax homogenizer (It is a digital homogenizer of the brand IKA T 18 ULTRA-TURRAX, China, at a speed of 11,000 rpm/min) until a homogeneous spore suspension was obtained.

Drops of the spore suspension were spread onto PDA (potato dextrose agar) supplemented with actidione (0.5 g/L) and gentamycine (2 mL/L). To prepare potato extract, the process first involves washing and cutting 200 g of potatoes, then boiling them in 1000 mL of distilled water for an hour. Once boiling is complete, the mixture is filtered to obtain the filtrate. To complete the filtrate, distilled water is added until the total volume reaches 1000 mL. Glucose is then added and the pH adjusted to suit the experimental requirements. To solidify the culture medium, 20 g of agar is added and carefully dissolved under heat. The medium is then sterilized by autoclaving at 120 °C for 20 min. After autoclaving, the antibiotic is added. The cultures were incubated in darkness at a temperature of 25 °C until the growth of mycelium. The pure mycelium was weekly transferred onto fresh PDA and conserved at 4 °C.

### 2.4. Molecular Characterization of the Mycelium

Extraction of genomic DNA from pure mycelium was performed using a DNA kit, following the manufacturer’s protocol. Internal transcribed spacer (ITS) region primers were used for the DNA amplification. The reaction mixture contained the following volumes: 2 μL genomic DNA extracts, 6 μL 5X (The multiplication sign X has been used to represent the PCR mix concentration). PCR mix, 1 μL primer forward, 1 μL primer reverse, and H_2_O qsp 30 μL. The thermal cycler was programmed as follows: an initial denaturation step at 94 °C for 12 min followed by 35 cycles of denaturation at 96 °C for 20 s, a hybridization phase at 56 °C for 20 s, an extension phase at 72 °C for 1 min, and a final extension at 72 °C for 5 min. A negative control (H_2_O) was amplified under the same conditions as DNA.

We used the primers ITS1 and ITS2:

ITS1 (Forward): 5′-TCCGTAGGTGAACCTGCGG-3′
ITS2 (Reverse): 5′-GCATATCAATAAGCGGAGGA-3′

Sequencing of reactions was performed on a 96 capillary ABI3730XL sequencer at the Biofidal Laboratory in Lyon, France.

The nucleotide sequences of the mycelium obtained in this study were deposited into the GenBank database of NCBI. Following the acquisition and rectification of the sequences through the interpretation of electropherograms, a BLAST (Basic Local Alignment and Search Tool) search was conducted on the internet (http://www.ncbi.nlm.nih.gov, accessed on 18 November 2020) to compare the mycelium sequence with those found in databases and to align them [22]. For a given alignment, an associated score is consistently present. Alignment programs consistently strive to maximize this score. It can be presumed that the greater the score, the higher the similarity. However, this rationale holds true within a homogeneous family where all sequences possess roughly the same length. In this scenario, a higher score denotes a stronger resemblance, yet it does not necessarily denote significance [23].

For the analyzed mushroom, an emphasis on percentages of identity or similarity between two sequences will be made instead of relying on scores. Our criteria for likelihood are as follows: if the nearest sequences obtained from databases showcase a similarity of 97–100%, and if these sequences correspond to the identical genus and species names, then the outcome derived aligns with our initial identification based on morpho-anatomical criteria for the genus and species [24]. The alignment of sequences was executed utilizing the MUSCLE algorithm within MEGA-X. Subsequently, a phylogenetic tree emerged from the final alignment using the implemented maximum likelihood program within MEGA-X. *Agaricus* sp. (AM930985.1) was introduced as an outgroup species within the context of the phylogenetic tree.

### 2.5. Effect of Physicochemical Factors on the Mycelial Growth

Ref. [25] described the methods used in mycelial culture. Eight different temperatures (15, 17, 19, 21, 23, 25, 28, and 30 °C), six pH values (4, 5, 5.6, 6, 7, and 8), and six different humidity values (14%, 50%, 74%, 80%, 95%, and 100%) were tested in order to determine the optimal mycelial culture. The effect of light was studied by subjecting inoculated Petri dishes to an intensity of 500 lux for durations of 5, 10, 15, 20, and 24 h. In each experiment, a mycelial fragment with a diameter of 1cm from a 10-day-old preculture was placed in the center of PDA medium in Petri dishes with a diameter of 9 cm. Following a 10-day incubation period under each physicochemical condition, the growth of mycelium was assessed by measuring the diameter of the fungal colony in centimeters. Each test was conducted in quadruplicate. The data evaluation was conducted over a 15-day growth period.

### 2.6. Primordia of Lepista

According to [26], the mycelium was cultivated in pots containing wheat grains. The wheat grains were rinsed and then immersed in water for 24 h. Subsequently, the grains were cooked for approximately 20 min. After cooking, excess moisture on the surface of the grains was drained. The grains were then placed in 500 mL flasks, with a quantity of 250 g per flask, and subjected to autoclave sterilization. Following sterilization, the grains were rehydrated using sterile distilled water. Next, the *Lepista sordida* mycelium was introduced. Finally, the flasks were incubated at 25 °C for 15 days to allow for colonization by the mycelium.

A light intensity of 500 lux was exposed to the wheat-invaded jars for 12 days. This period was sufficient for the appearance primordia [27], which were collected and examined with optical microscope. All experiments were conducted in quadruplicate for each value. The results were expressed as mean ± standard deviation. Moisture, ash content, fat, protein, and total carbohydrates were calculated.

### 2.7. Nutritional Value of Harvested Lepista Sporophores

The nutritional components (moisture, ash, proteins, fat, and carbohydrates) of the freshly harvested sporophores of the wild edible mushroom were analyzed using various methods. It is important to take into account the condition of the mushroom (fresh, dried, or freeze-dried), as well as its color and aroma. All the analyses were carried out in triplicate.

The moisture content method was based on the principle of measuring the weight loss of the sample after placing in an oven at 105 °C overnight until a constant weight was reached [28].

The ash content was determined by incinerating the sporophores of the mushrooms in a muffle furnace at 550 °C for 24 h. After cooling, the ashes were placed in adesiccators [28].

The protein content in fresh sporophores was determined by calculating the total nitrogen content using the Kjeldahl method and applying the conversion factor (N × 4.38). The nitrogen was highlighted by subjecting a flask to mineralization at 105 °C, and the completion of mineralization was indicated by a pale green color. After cooling, the analysis was carried out using the Kjeldahl apparatus [29].

The fat content was determined using hexane as a solvent in a Soxhlet extractor. After the hexane was evaporated and the capsule dried in an oven at 105 °C for 30 min, the difference in weight provided the fat content of the sample [30].

For the measurement of carbohydrates, the phenol–sulfuric acid method was employed to determine the sugar content in the mushroom sample. This method is a simple and rapid colorimetric technique [31].

### 2.8. Statistical Analysis

Statistical analysis was performed using the Graph Pad Prism 7 software program. The data were analyzed using a one-way analysis of variance (ANOVA). To detect the statistical significance of differences (*p* < 0.05) between means, the Tukey test was performed.

## 3. Results

### 3.1. Mushroom Characteristics

**Figure 1:**
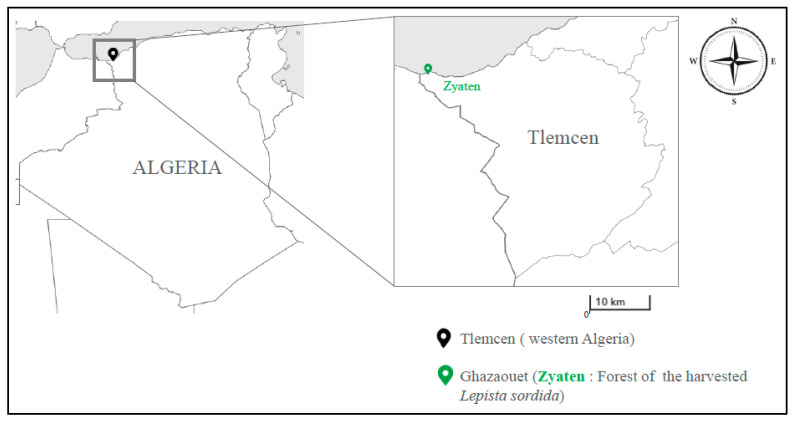
Localization of the harvested mushroom (illustration source: https://mapmaker.nationalgeographic.org/, accessed on 18 November 2020).

**Figure 2:**
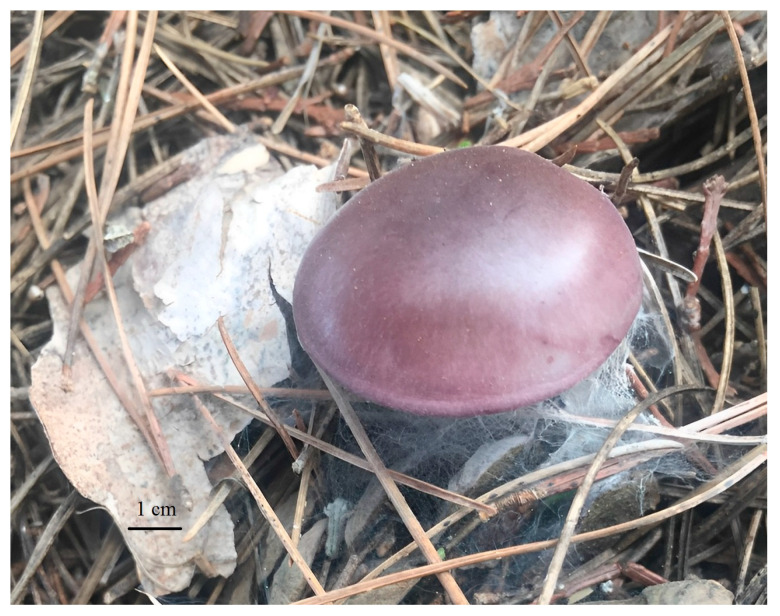
General Aspect of the edible wild mushroom *Lepista sordida* in nature.

This is the first observation of *Lepista sordida*, which is considered wild saprophytic edible mushroom, in western Algeria (Figure 1). The sporophores of *Lepista sordida* (Figure 2), growing on dead leaves, were collected in Tlemcen (Ghazaouet, in western Algeria). The characterization of the mushroom was initially based on macroscopic features and habitats.

**Figure 3:**
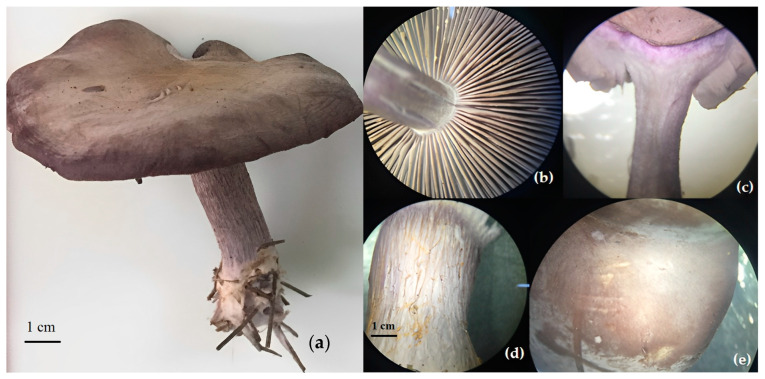
Morphological characteristics of *Lepista* harvested in western Algeria. (**a**): entire sporophore, (**b**): gills details, (**c**): cross-section cut in the sporophore, (**d**): stem pileus texture, (**e**): cap texture.

*Lepista sordida* is a small-sized mushroom, measuring between 4 and 8 cm (Figure 3). It is a violet color, sometimes with brownish reflections. Its cap is hygrophanous, meaning it becomes translucent with water. Initially convex, it gradually flattens while retaining a central bump. The cap surface is smooth, the gills are close together, and the stem is short and curved, with a cottony base due to remnants of mycelium. The flesh is smooth, firm, and whitish with violet hues. This mushroom emits a strong fungal odor and has a pleasantly sweet taste. All these morophological characteristics were reported by [32,33], confirming the identity of the harvested mushroom as *Lepista sordida.*

**Figure 4:**
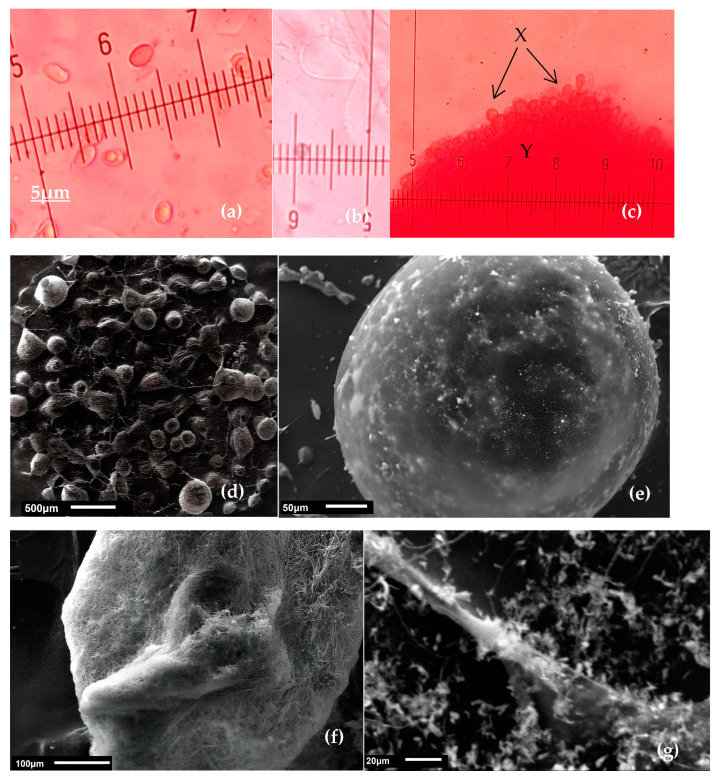
Microscopic characterization of *Lepista sordida*: (**a**) basidiospores 2.5–3 × 1–1.5 µm (Gr × 100); (**b**) basidia; (**c**) interwoven pileipellis (X: cellular subhymenium, Y: immature basidia); (**d**) distant view of spores, SEM × 27; (**e**) SEM of spore × 270; (**f**) anastomosis hook, SEM × 200; (**g**) thallus ramification SEM × 650.

A Congo red staining method was used to observe the basidiospores of *Lepista sordida* with an optical microscope (Figure 4). Basidiospores observed with scanning electron microscopy had a cylindrical shape with irregular and verruculose ornamentations. Most of the observed basidiospores had a large lipid body, absence of acid vesicles, and a nucleus located at the periphery. Germinated spores appeared as deflated balloons, emptied of their contents. The spores of *Lepista sordida* developed hyphae.

### 3.2. Molecular and Phylogenetic Analyses

**Figure 5:**
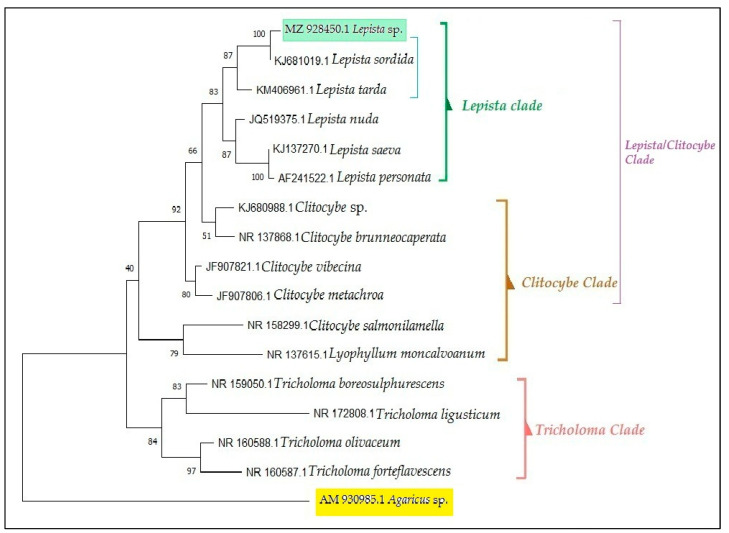
Phylogenetic tree of the edible wild mushroom collected in western Algeria (indicated in green). The species marked in yellow represents the outgroup used for tree construction. The results of the phylogenetic analysis are represented by colors: green for the *Lepista clade*, brown for the *Clitocybe clade*, and pink for the *Tricholoma clade*. The blue color indicates that *Lepista sordida* and *Lepista tarda* belong to the same clade, as mentioned in the text.

The ITS nucleotide sequences obtained from the mycelium of Lepista sordida allowed us to identify it. Molecular biology techniques were applied to the mushroom’s mycelium by sequencing the ITS region of the DNA, which is the most commonly used molecular marker for fungal species [34]. The nucleotide sequences of the mycelium obtained in this study were deposited into the GenBank database of NCBI under the accession number: MZ928450.1. Next, the sequences of the closest related species were extracted using the BLAST method from the GenBank database to find the best similarities with other related ITS sequences in the database. The adaptation of the kit protocol in the laboratory enabled optimal DNA extraction. Consequently, the DNA of the studied mycelial fungus could be extracted and amplified. The sequence of the strain MZ928450.1 of *Lepista* sp. turned out to be identical to the sequence of *Lepista sordida* KJ681019.1 (Figure 5). In the phylogenetic tree constructed using the ITS DNA sequence (Figure 5), *Lepista sordida* MZ928450.1 was clustered within a clade alongside other species from the same genus, *Lepista sordida*. These species include *Lepista tarda*, *Lepista nuda*, *Lepista saeva*, and *Lepista personata*, showing an approximate dissimilarity distance of 0.0124. The *Lepista sordida* clade can be grouped in the same clade as *Lepista tarda* and closely associated with certain *Clitocybe* sp. (*Clitocybe brunneocaperata*).

The evolutionary lineage was deduced using the maximum likelihood technique along with the Jukes–Cantor model [35]. The tree presenting the highest log likelihood (−2711.16) has been depicted, with the percentage representing the instances where the related taxa have clustered together shown alongside the branches. The initial tree(s) for the exploratory search were generated automatically by employing neighbor-join and BioNJ algorithms on a pairwise distances matrix, which was derived using the Jukes–Cantor model. Subsequently, the topology with the most superior log likelihood value was selected. The tree is drawn proportionally, with the extent of branches measured in substitutions per site. This analysis encompassed a total of 17 nucleotide sequences. All sites containing gaps and incomplete data were excluded (using the complete deletion option). The final dataset consisted of 566 positions. Evolutionary investigations were carried out using MEGA X [36].

### 3.3. Effect of Physicochemical Factors on the Mycelial Growth of Lepista sordida

**Figure 6:**
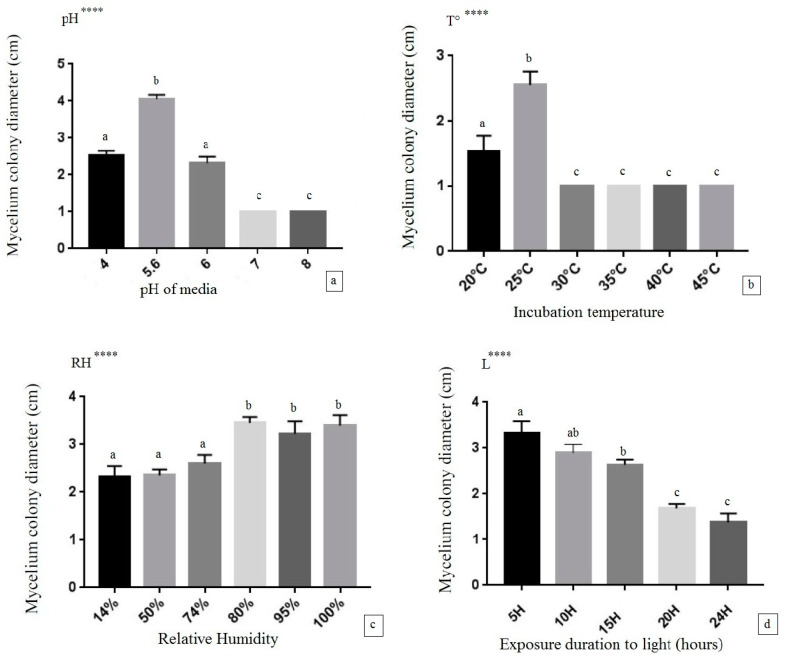
Graphical representations of *Lepista sordida* mycelial growth in different physicochemical parameters: (**a**) pH, (**b**) temperature, (**c**) relative humidity, (**d**) light exposure duration. One-way ANOVA significance levels: ns: not significant ****: *p* < 0.0001. Different letters (a, b, c) indicate significant differences from each other (*p* < 0.05) according to the Tukey test.

The effects of physicochemical parameters in the culture, such as pH, temperature, relative humidity, and light, were investigated on the mycelial growth of *Lepista sordida* (Figure 6).

The results revealed that variations in the different physicochemical parameters significantly influenced mycelial growth. The optimal pH for mycelial growth was 5.6, with slight growth observed between pH 4 and 6 (acidic); no growth was observed at pH 7 and 8. The mycelial growth of *Lepista sordida* at 25 °C was significantly higher (*p* ≤ 0.0001) compared to a slight growth observed at 20 °C. However, no growth was observed at 30, 35, 40, and 45 °C. Relative humidity (RH) was a significantly influential factor (*p* < 0.0001). The results demonstrated that there was no difference among humidity levels at 80, 95, and 100%. The one-way analysis of variance (ANOVA) conducted on the mycelial growth of *Lepista sordida* indicated that the light factor significantly influenced the mycelial growth.

### 3.4. Primordia of Lepista

Mushroom spawn production is one of the most critical steps in mushroom cultivation used to propagate a sterile culture of mycelium. The culture can be produced under laboratory conditions from sporophores spores [37].

**Figure 7:**
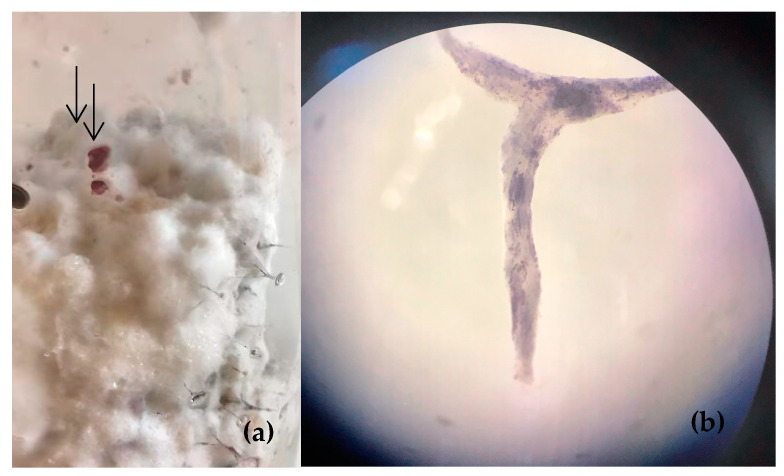
Mycelial growth of *Lepista sordida* primordia (**a**): appearance of wheat grains invaded by *Lepista* mycelium with purple hyphae (arrow). (**b**): Observation of purple filaments under a magnifying glass.

The different phases of *Lepista sordida* cultivation (Figure 7) were studied, including mycelial colonization and primordia initiation. The results revealed that growth was achieved within 21 days. The trial for sporophore production in *Lepista sordida* showed promising results at the primordia stage rather than at the sporophore stage. Primordia represent the preliminary stages of sporophore formation, and the experiment was repeated multiple times to obtain reliable results.

### 3.5. Nutritional Value of the Harvested Sporophores

The nutritional value of wild-harvested sporophores from *Lepista sordida* was determined. The obtained results are recorded in Table 1.

## 4. Discussion

Mushrooms can be classified according to their edibility, which refers to their suitability for human consumption. Macroscopic descriptions of specimens are based on fresh sporophores as well as mature and young sporophores [38]. The morphological characteristics of *Lepista sordida* include the deep purple or lilac-brown color of the cap and the pink spore print. These characteristics are difficult to distinguish from the well-known *Lepista nuda*, which also has a violet color at its mature stage [15].

The results obtained regarding the characteristics of the spores, whose observations were carried out using scanning electron microscopy (SEM), reveal similarities with the basidiospores of *Lepista sordida*, as studied by [39]. This confirms the effectiveness of using scanning electron microscopy for taxonomic applications, such as distinguishing this species from other similar fungi. These findings pave the way for future studies on the biology of *Lepista sordida*. As a result, this has potentially led to identification errors, which are common both between and within *Lepista* species [14]. Fungal spores are individual cells, each containing a single haploid nucleus. During germination, they produce homokaryotic hyphae, with each hyphal cell containing a nucleus. The role of nuclei in basidiospores is crucial for their life cycle and reproduction, which is why they are referred to as reproductive spores [40]. Observation under the optical microscope played a crucial role in characterization by providing a detailed description of the spores and mycelium of *Lepista sordida*. These results show similarities with the observations from the study conducted by [33]. Therefore, this study aimed to contribute to the assessment of *Lepista sordida* based on morphological and molecular evidence in Indonesia. The phylogenetic tree established in this study has provided a framework for understanding the evolutionary history of closely related species within the *Lepista* clade and their relationship with other clades, such as the *Clitocybe* and *Tricholoma* clades. Although certain morphological characteristics differ among these groups, the clades can be identified using morphological, molecular, or a combination of both types of data. The phylogenetic analysis of ITS sequences, as recorded by [33], has also demonstrated that the *Lepista sordida* clade can be grouped in the same clade as *Lepista tarda* and closely associated with certain species of *Tricholoma* sp.

*Lepista sordida* is consumed in China due to its pleasant color and aroma, making it a favored edible mushroom [41]. *Lepista* species are saprophytic and can be found on the soil or humus, exclusively growing on the ground or litter rather than on wood. The boundaries between the *Lepista* and *Clitocybe* genera are indistinct; under the microscope, *Lepista* spores exhibit a verrucose to echinulate surface, while *Clitocybe* spores are smooth [42]. *Lepista sordida* is considered to be a valuable source of carbohydrates, essential amino acids, and dietary fibers [43].

Mycelial growth is considered to be the vegetative phase of the growth cycle. During this phase, the mycelium tries to colonize and decompose any available substrate. It exudes enzymes (which promote decomposition) onto the substrate surface, and the nutrients that become available are absorbed back into the mycelial network. This mycelial network will continue to expand until it no longer has a fresh nutrient source to colonize or until it encounters another competitive organism, such as a contaminant in the culture [44].

The mycelium of *Lepista sordida* contains intracellular polysaccharides [45]. Thus, this mushroom exhibits excellent free radical scavenging activity, which can be attributed to the presence of a significant amount of antioxidant compounds such as phenols, flavonoids, and ascorbic acid. These compounds could be explored as potential dietary supplements to mitigate age-related diseases in humans and delay aging [46].

The growth medium is the most important factor in mushroom production as it is considered the source of nutrients necessary for the mycelium’s growth. The commonly used and most effective media for promoting fungal culture growth are malt-extract agar (MEA), given preference first, followed by PDA (potato dextrose agar) in second place [47,48].

According to [49], temperature is a highly significant environmental factor for mushroom mycelium growth. The variation in physicochemical parameters during the cultivation process impacts the mycelial growth of mushrooms in a culture medium. Key factors influencing mycelial growth and the development of fruiting bodies include pH, temperature, humidity, light, and oxygen availability [50].

These parameters represent standard optimal conditions for the majority of fungal cultures. The impact of physicochemical factors (including pH, temperature, light, and humidity) on the cultivation of edible mushrooms has been extensively examined, encompassing a broad spectrum of variations for the *Pleurotus ostreatus* species. The highest values were recorded at a pH of 5.6, temperature of 25 °C, and relative humidity of 80% [51]; these results are similar for the mycelial culture of *Lepista sordida*.

The purpose of the mycelium-coated grain is to rapidly colonize the specific bulk growth substrate. The quality of the spawn is essential for successful mushroom production, and it must be prepared under sterile conditions to reduce substrate contamination. The study by [52] was conducted with the aim of improving spawn quality and developing new production techniques. This study specifically explored spawn preparation on different types of grains and straw to enhance mushroom cultivation yields.

The results published by [48] demonstrated that the substrate containing wheat grains exhibited a higher mycelial density compared to other substrates. Subsequently, the mycelial growth rate was monitored on barley grains, followed by oat grains. No significant difference was observed between substrates based on rye, oat, and barley grains in terms of mycelial extension. The results obtained from the light test confirm the theory of [53], showing that light has been considered a factor that weakens mycelial growth, although [54] found that blue light suppressed the growth of *Pleurotus ostreatus* mycelium.

The present study aimed to evaluate the chemical composition of the extract obtained after analyzing the sporophores of *Lepista sordida* in terms of nutritional richness. The results clearly demonstrated that these mushrooms have a high nutritional value, making them extremely promising in addressing mineral and vitamin deficiencies in the diet. *Lepista sordida* is recognized for its high nutritional value and medicinal properties. It is a fungus rich in polysaccharides that possess physicochemical properties [55,56].

Cultivated mycelium and cultivated sporophores can indeed exhibit distinct compositions compared to natural fruiting bodies [57]. The mycelium represents the vegetative part of the fungus containing higher levels of certain secondary metabolites. On the other hand, sporophores are the reproductive structures, including the mushrooms themselves, and their composition can vary based on factors such as growth conditions, nutrient availability, and developmental stage [58]. Sometimes, we find similar compositions; for example, primordia contain hericenes, which are metabolic compounds. These compounds are also present in the mycelium and sporophores, encouraging the exploration of dietary supplements developed from these bioactive metabolites [59]. Mushrooms are emerging as a promising natural resource for functional and therapeutic foods. The Lepista genus is being explored for its nutritional and medicinal attributes, offering prospects for new bioactive products. This review highlights its potential as an effective treatment [60].

## 5. Conclusions

When a mushroom picker harvests an unknown specimen, they start by conducting an initial macroscopic identification, examining the shape and color of the cap, stem, gills, and flesh. Additionally, the texture, a significant macroscopic aspect in the identification of basidiomycete mushrooms, is evaluated. The smell is also taken into consideration. Subsequently, the observation of gills under the cap, including their color, spacing, attachment to the stem, and the presence of a veil, can provide additional clues for identification. Microscopic characterization involves observing different parts of the mushroom using an optical microscope, as well as using scanning electron microscopy (SEM). Furthermore, molecular characterization allows for DNA analysis, providing precise and reliable identification. Regarding the cultivation of *Lepista sordida* for optimal growth, standard conditions are necessary, similar to those required for the majority of mushroom cultures.

The nutritional analysis of *Lepista sordida* revealed varying levels of moisture, ash, fats, proteins, and carbohydrates. The results clearly demonstrated that this edible mushroom exhibits a high nutritional value, rendering it highly promising for addressing deficiencies in minerals and vitamins in the diet.

## Figures and Tables

**Table 1:** Nutritional value of the wild edible mushroom sporophores cultivated in grow room.

Moisture (%)	Ash (%)	Fat (%)	Protein (%)	Total Carbohydrates (%)
67.23 ± 1.90	9.35 ± 0.66	3.25 ± 0.24	17.22 ± 0.38	63.83 ± 1.23
